# Unsupervised cross-lingual model transfer for named entity recognition with contextualized word representations

**DOI:** 10.1371/journal.pone.0257230

**Published:** 2021-09-21

**Authors:** Huijiong Yan, Tao Qian, Liang Xie, Shanguang Chen

**Affiliations:** 1 College of Mechanical and Vehicle Engineering, Taiyuan University of Technology, Taiyuan, China; 2 Defense Innovation Institute, Academy of Military Sciences (AMS), Beijing, China; 3 National Key Laboratory of Human Factors Engineering, China Astronaut Research and Training Center, Beijing, China; 4 School of Computer Science and Technology, Hubei University of Science and Technology, Xianning, China; National University of Singapore, SINGAPORE

## Abstract

Named entity recognition (NER) is one fundamental task in the natural language processing (NLP) community. Supervised neural network models based on contextualized word representations can achieve highly-competitive performance, which requires a large-scale manually-annotated corpus for training. While for the resource-scarce languages, the construction of such as corpus is always expensive and time-consuming. Thus, unsupervised cross-lingual transfer is one good solution to address the problem. In this work, we investigate the unsupervised cross-lingual NER with model transfer based on contextualized word representations, which greatly advances the cross-lingual NER performance. We study several model transfer settings of the unsupervised cross-lingual NER, including (1) different types of the pretrained transformer-based language models as input, (2) the exploration strategies of the multilingual contextualized word representations, and (3) multi-source adaption. In particular, we propose an adapter-based word representation method combining with parameter generation network (PGN) better to capture the relationship between the source and target languages. We conduct experiments on a benchmark ConLL dataset involving four languages to simulate the cross-lingual setting. Results show that we can obtain highly-competitive performance by cross-lingual model transfer. In particular, our proposed adapter-based PGN model can lead to significant improvements for cross-lingual NER.

## Introduction

Named entity recognition (NER) aims to extract named entities and meanwhile identify their semantic types (e.g., person, organization and location) from text, which is one of the fundamental tasks in natural language processing (NLP) [1]. The task can be beneficial for a range of applications, including relation extraction [2], coreference resolution [3] and question answering [4], as the extracted named entities are critical elements for these applications.

**Fig 1:**
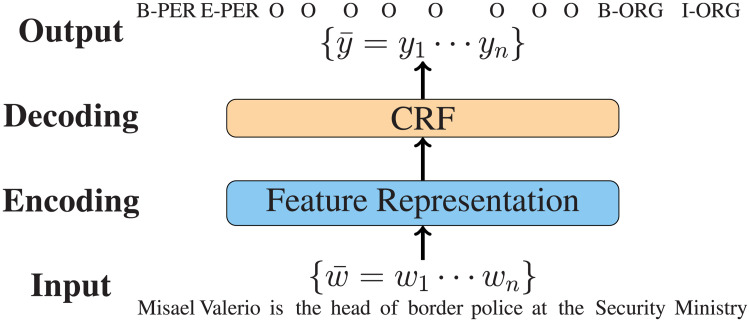
An example of neural NER with sequence labeling.

NER is generally treated as a sequence labeling problem by word-level tagging, where the tags are defined according to the entity boundary information [5]. Fig 1 shows one example for NER modeling. Conditional random fields (CRF) models with neural networks have achieved state-of-the-art performance [6, 7]. In particular, equipped with contextualized word-level neural representations such as BERT [8], neural NER systems can reach a performance over 92% by the F-measure on the benchmark ConLL03 English dataset, close to 2% increases over the previous systems [6, 9, 10].

All these successes are based on supervised setting, assuming that a large-scale manually-constructed high-quality corpus is available for model training. However, it is not always the setting in practical, especially for the resource-scarce languages, where there exists no training corpus to learn such a supervised model. According to the official statistics, there are over 7,000 languages today, and most of them do not have any annotated corpus for NER.

The model transfer is one mainstream method for unsupervised cross-lingual adaption [11–13], which builds cross-lingual models on language-independent features such as cross-lingual word representations, and thus the learned models can be applicable to target languages directly. The method has received great attention as it is quite straightforward and easy to follow. Under the neural setting, previous cross-lingual NER studies are mostly focusing on multilingual word embeddings [14], or a simple exploration of mBERT to another line of transfer strategies [15, 16].

In this work, we present the first work to study cross-lingual model transfer with contextualized word representations for NER comprehensively. Here we mainly focus on mBERT and XLM, which are two widely-adopted multilingual contextualized word representations based on the transformer network. Our first goal is to compare the two kinds of word representations, and meanwhile investigate fine-tuning and feature-based methods of exploiting mBERT and XLM [8]. Fine-tuning is the standard strategy because of its high performance, whereas the feature-based strategy by frozen the mBERT or XLM parameters is much more parameter-efficient. Here, we adopt the adapter mechanism for the feature-based strategy in order to make the two strategies comparable in performance. Finally, we study single-source and multi-source model transfer, and propose a novel model based on parameter generation network (PGN) [17] to better capture the differences between the source and target languages.

We conduct experiments on the benchmark ConLL dataset to evaluate our models, which includes four languages: English, Spanish, German and Dutch, respectively. The languages are used to simulate the resource-scarce languages, where only the test dataset is available when one is selected as the targeted language. First, we can find that XLM can achieve better performance than mBERT with fair comparisons as a whole. Second, the adapter enhanced feature-based method is one good alternative for model transfer, which achieves very competitive performance with much less learned parameters. Third, multi-source transfer can help the target language greatly, leading to averaged improvements by 2.79 points compared with the best-reported bilingual transfer.

In summary, our major contributions in this article are three folds. (1) We present the first comprehensive work to investigate cross-lingual NER by using model transfer with contextualized word representations, including comparisons between different multilingual contextualized word representations, different exploration methods of word representations as well as multi-source transfer. (2) We are the first work of exploiting the adapter module and parameter generation network to enhance multilingual contextualized word representations for unsupervised cross-lingual model transfer. (3) We empirically evaluate the model transfer method with various settings for cross-lingual NER. The codes and related data are released publicly available at https://github.com/qtxcm/UCT-NER under Apache License 2.0.

## Method

Given an input sentence, the goal of NER is to identify all entities with specific named types, such as Person, Organization, Location, and etc. The standard sequence labeling architecture is widely exploited to formalize the task, which transforms entities/non-entities into character-level boundary labels by using the BIO or BIOES schema. Here we adopt the the BIOES schema, where each sentential word is labeled as either “O”(non-entity words), “B-XX” (the beginning word of an entity with type “XX”), “E-XX” (the end word of an entity with type “XX”), “I-XX” (the middle word of an entity with type “XX”), or “S-XX” (a single-word entity with type “XX”). Notice that the overlapped or discontinuous entitles are not considered in this work.

### The model

We adopt BERT-BiLSTM-CRF as the main model structure for our NER task, which can achieve state-of-the-art performance for NER [9]. The model consists of four components: (1) word representation, (2) Encoding, (3) CRF decoding, and (4) training, and particularly for word representation, we consider the fine-tuning and feature-based strategies for external pretrained parameters. Fig 1 shows the overall architecture by an example. Here we introduce the model in detail.

#### Word representation

The word representation is the key to cross-lingual NER of model transfer, which serves as the primary bridge to adapt to multiple languages. For a given sentence $\bar{w}=w_{1}\cdots w_{n}$ (i.e., *n* indicates the sentence length), we first convert it into sequential hidden vectors by using pretrained contextualized language model:

$$\begin{array}{c} \begin{array}{c} e_{1}\cdots e_{n}=T\text{RANSFORMER}PLM\left(\bar{w}\right). \end{array} \end{array}$$ (1)

**Fig 2:**
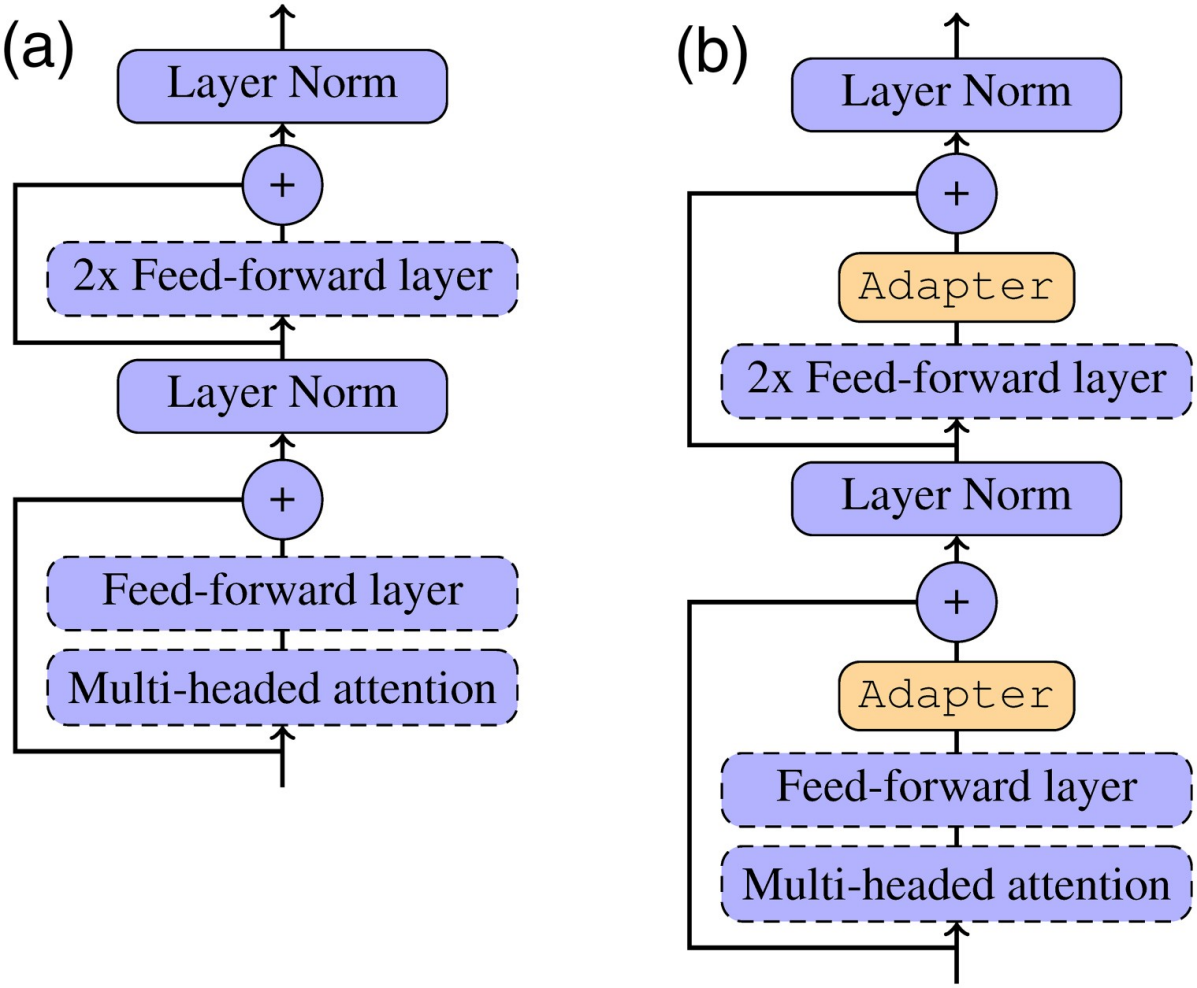
The internal structure of a one-layer transformer. (a) Standard Transformer. (b) Transformer with Adapters.

Here we only exploit the pretrained transformed-based language models. The overall networks of these pretrained models are stacked by several standard transformer layers, as shown in Fig 2a. Each transformer submodule [18] is organized as follows:

$$\begin{array}{c} \begin{array}{cc} & q_{i}^{1\cdots H},k_{i}^{1\cdots H},v_{i}^{1\cdots H}=S\text{PLIT}\left(W_{q}x_{i},W_{k}x_{i},W_{v}x_{i}\right),\\ & a_{i,j}^{l}=S\text{OFTMAX}(\frac{T\text{RANSPOSE}(q_{i}^{l})k_{j}^{l}}{\sqrt{\text{head}}}),\;\;\;\;\;l\in \left[1,H\right],\\ & r_{i}=C\text{ONCAT}\left(\sum_{j=1}^{n}a_{i,j}^{1}v_{i}^{1},\cdots ,\sum_{j=1}^{n}a_{i,j}^{H}v_{i}^{H}\right),\\ & y_{i}=L\text{AYER}N\text{OR}\left(x_{i}+r_{i}\right),\\ & f_{i}=W_{2}R\text{ELU}\left(W_{1}y_{i}+b_{1}\right)+b_{2},\\ & z_{i}=L\text{AYER}N\text{orm}\left(y_{i}+f_{i}\right), \end{array} \end{array}$$ (2)

where $\bar{x}=x_{1}\ldots x_{n}$ is the input, which is the word and positional embeddings at the first layer and the previous layer outputs for the other layers, and $\bar{z}=z_{1}\ldots z_{n}$.

In more detail, Split divides a vector into H components, and following, self-attention aggregations are performed for the H components, respectively. Then, the updated hidden vectors after these individual self-attentions are concatenated. The above process is referred to as multi-head self-attention. After the multi-head attention, a layer normalization (i.e., LayerNorm) is used to regularize the original input ***x***_*i*_ and the attention output ***r***_*i*_. Finally, a two-layer feed-forward layer is executed, followed by another layer normalization, producing the one-layer transformer output ***z***_*i*_.

In this work, we investigate two multilingual word representations: mBERT and XLM [15, 16], which are both pretrained language models based on the transformer network. Although the BiLSTM-based ELMo is also a widely-studied language model, we ignore it here because of its lower performance for cross-lingual model transfer. In addition, note that mBERT or XLM leads to subword-level representations, which include inconsistencies with our desired word-level outputs, as one full word might be decomposed to several subwords in mBERT and XLM. Here we use the averaged pooling over all the covered subwords for the representation of a full word. Thus, we can finally obtain a sequence of word-level representations $\bar{e}=e_{1}\ldots e_{n}$ as shown in Eq 1 for NER.

#### Fine-tuning or feature-based

Transformer-based language model pretraining has achieved state-of-the-art results on a wide range of NLP tasks [8]. It accepts full sentences as inputs, outputting contextualized word representations based on a well-pretrained bidirectional Transformer. Fine-tuning is the standard exploration for such pretrained language models. Concretely, the last-layer vectorial output is exploited for feature extraction, and all the internal parameters are fine-tuned along with our NER objective. It is the standard strategy for using transformer-based language models, and can bring great successes for a number of NLP tasks.

Although fine-tuning can achieve remarkable performances [8], the strategy may suffer from the parameter inefficiency problem, where a newly-trained model would introduce a new copy of mBERT or XLM weights (i.e., consuming 110M parameters). This process may lead to great inconveniences in real scenarios which involve multiple NLP tasks and model ensembles, since each model keeps a different copy of BERT weights. Thus, it is highly meaningful to study the feature-based method which freezes the parameters of mBERT or XLM during training. In this way, we can preserve a shared BERT across different NLP models. Here we follow the above observation, investigating the feature-based method of freezing the internal parameters of the large pretrained language models such as mBERT or XLM.

The preliminary experiments show that direct feature extraction from mBERT or XLM leads to significant decreases compared with fine-tuning. To reduce the gap, we exploit the adapter mechanism [19, 20], applying it to the unit transformer layers of mBERT or XLM. The overall process of one adapter can be formalized as follows:

$$\begin{array}{c} \begin{array}{cc} & \text{down-proj:}\;\;\;\;h_{\text{mid}}=\text{GELU}\left(W_{\text{down}}h_{\text{in}}+b_{\text{down}}\right),\\ & \text{up-proj:}\;\;\;\;\;\;\;\;\;h_{\text{out}}=W_{\text{up}}h_{\text{mid}}+b_{\text{up}}+h_{\text{in}}, \end{array} \end{array}$$ (3)

where ***W***_down_, ***W***_up_, ***b***_down_ and ***b***_up_ are model parameters, which are much smaller than those of transformer in scale, and the dimension size of ***h***_mid_ is also smaller than that of the corresponding transformer dimension. The dimension sizes of ***h***_in_ and ***h***_out_ are consistent with the corresponding transformer.

Fig 2b shows the differences between the standard transformer and the transformer with adapters. For each transformer, we insert the adapters before the two layer normalization layers [20]:

$$\begin{array}{c} \begin{array}{cc} & q_{i}^{1\cdots H},k_{i}^{1\cdots H},v_{i}^{1\cdots H}=S\text{PLIT}\left(W_{q}x_{i},W_{k}x_{i},W_{v}x_{i}\right),\\ & a_{i,j}^{l}=S\text{OFTMAX}(\frac{T\text{RANSPOSE}(q_{i}^{l})k_{j}^{l}}{\sqrt{\text{head}}}),\;\;\;\;\;l\in \left[1,H\right],\\ & r_{i}=C\text{ONCAT}\left(\sum_{j=1}^{n}a_{i,j}^{1}v_{i}^{1},\cdots ,\sum_{j=1}^{n}a_{i,j}^{H}v_{i}^{H}\right),\\ & \begin{array}{c} r_{i}^{'}=A\text{DAPTER}\left(r_{i}\right) \end{array},\\ & y_{i}=L\text{AYER}N\text{ORMS}\left(x_{i}+r_{i}^{'}\right),\\ & f_{i}=W_{2}R\text{elu}\left(W_{1}y_{i}+b_{1}\right)+b_{2},\\ & \begin{array}{c} f_{i}^{'}=A\text{DAPTER}\left(f_{i}\right) \end{array},\\ & z_{i}=L\text{AYER}N\text{ORMS}\left(y_{i}+f_{i}^{'}\right), \end{array} \end{array}$$ (4)

Note that we only tune the weights of the adapter modules during training, keeping all parameters of the pretrained language models fixed.

#### Encoding

We use a bidirectional LSTM layer to further abstract the final hidden features for our task [21], where the process can be formalized as:

$$\begin{array}{c} \begin{array}{cc} h_{1}\cdots h_{n} & =BI\text{LSTM}(\left[e_{1}\cdots e_{n}\right]) \end{array} \end{array}$$ (5)

where $\bar{h}=h_{1}\ldots h_{n}$ is the desired features for the next decoding.

Actually, the majority of previous studies show that mBERT or XLM with fine-tuning does not require any other extra encoding layers, and the output $\bar{e}=e_{1}\ldots e_{n}$ can be directly exploited for decoding with little loss in performance. Here, we insist on a BiLSTM encoding since the performance is more stable and slightly better after using it. In addition, the BiLSTM encoding can be effective for the feature-based methods.

#### Decoding

CRF has been a standard decoding strategy for sequence labeling [22]. First, a linear feed-forward transformation layer is exploited to calculate the initial label scores. Then, a label transition matrix ***T*** is used to model first-order Markov chains. Let $\bar{y}=y_{1}\ldots y_{n}$ be any output label sequence, its score $s(\bar{y}|\bar{x})$ can be computed by:

$$\begin{array}{c} \begin{array}{cc} & o_{i}=Wh_{i}+b\\ & s\left(\bar{y}|\bar{x}\right)=\sum_{i=1}^{n}\left(T_{y_{i-1},y_{i}}+o_{i}\left[y_{i}\right]\right) \end{array} \end{array}$$ (6)

where ***W***, ***b*** and ***T*** are the model parameters. The decoding aims to find the highest-scored $\bar{y}$, and here we utilize the Viterbi algorithm to achieve the goal.

#### Training

We exploit the sentence-level cross-entropy objective for our task training. Given a gold-standard training instance $\left(\bar{x},\bar{y}\right)$, we first compute the conditional probability $p(\bar{y}|\bar{x})$ based on the score function defined in Eq 6, and then apply a cross-entropy function to obtain the single instance loss:

$$\begin{array}{c} \begin{array}{cc} & p\left(\bar{y}|\bar{x}\right)=\frac{\exp(s(\bar{y}|\bar{x}))}{\sum_{\tilde{y}\in \tilde{Y}}\exp(s(\tilde{y}|\bar{x}))},\\ & L\left(\bar{x},\bar{y}\right)=-\log p\left(\bar{y}|\bar{x}\right), \end{array} \end{array}$$ (7)

where $\tilde{Y}$ denotes all possible candidate predictions for sentence $\bar{x}$.

### Cross-lingual model transfer

Based on the above model, we can achieve our cross-lingual model transfer by using multilingual word representations. Moreover, since no language-dependent feature is exploited in the NER model, we can use the model for any language in the world, treating all languages with no discrimination. Thus, we can exploit a training corpus of any language for a target language. In particular, if only one language is exploited for model training, we usually refer to it as single-source cross-lingual model transfer, whereas if multiple languages are exploited during training, we characterize it as multi-source cross-lingual model transfer.

In this work, we present a new method that enables sophisticated representations of input languages. By this method, we can capture the distances between different languages, which can better help the single-source and multi-source cross-lingual model transfer. The key idea is to use an embedding to denote each language, where several languages may have very similar embedding vectors and others are different. To use the embeddings, we exploit the parameter generate network (PGN) to enhance the word representations of our basic model.

We apply the PGN only to the adapter-based word representations, where the parameters inside pretrained language models are kept frozen. The main reason lies that it is difficult to integrate the language embeddings into the fine-tuned methods, which requires a re-pretraining for the language models by using a large-scale raw corpus. In contrast, only the adapter parameters are required to be tuned for the adapter-based method, which can be combined with PGN and learned from a distant task lightly.

**Fig 3:**
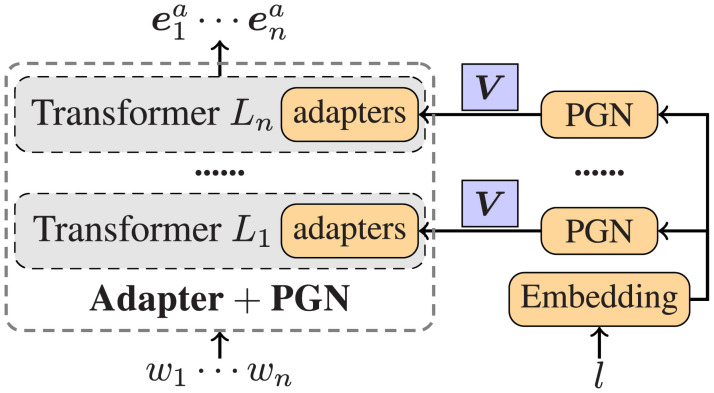
The pretrained transformer-based language model coupling with adapters and PGN.

Fig 3 shows the concrete network architecture of our adapter-based word representation with PGN. Formally, we collect all adapter parameters as a whole and pack them into a vector ***V***_*ada*_, and also the vector can be unpacked for the calculation of individual adapters. In the vanilla adapter model, ***V***_*ada*_ is shared by all languages with the same values, and after PGN is exploited, ***V***_*ada*_ is produced dynamically for each language by the following equation:

$$\begin{array}{c} V_{ada,l}=\Omega e_{l}, \end{array}$$ (8)

where ***V***_*ada*,*l*_ indicates the new adapter parameters for a given language *l*, **Ω** is one model parameter, and ***e***_*l*_ is the language embedding.

Since we do not have any target-language corpus to train our model, thus the determination of ***e***_*l*_ is difficult. Although we can learn the source language embeddings from the training corpus, the target language embedding is in no way to be obtained. Here we adopt a distant supervision method to pretrain the language embeddings, which is then fixed for our final NER model in the cross-lingual supervision. We exploit a parallel corpus to achieve the goal by a binary classification to judge if a pair of sentences of different languages are translations of each other. We simply use the adapter-based word representations to derive a sentence vector, and then diff the pairwise vectors for classification. The positive instances are sentences from the parallel corpus, while the negative instances are randomly sampled. In this way, we can obtain the language embeddings as well as initialized adapters in advance, which are then fed into our neural NER model, tuning only the parameter **Ω** continually.

## Experiments

### Experimental settings

#### Datasets

We construct a benchmark dataset by merging the CoNLL-2002 (Spanish and Dutch) and CoNLL-2003 (English and German) datasets [23, 24]. All corpora of the four languages are annotated with 4 entity types: PER, LOC, ORG, and MISC, and each language-specific dataset is split into training, development, and test sets. Table 1 shows the dataset statistics. We use the BIOES scheme to convert NER into the sequence labeling problem.

**Table 1:** Data statistics, where the number of sentences and entities are reported, and Devel indicates the development set.

Language	#Sentence			#Entity		
	Train	Devel	Test	Train	Devel	Test
English (en)	14,987	3,466	3,684	23,499	5,942	5,648
German (de)	12,705	3,068	3,160	11,851	4,833	3,673
Spanish (es)	8,323	1,915	1,517	18,798	4,351	3,558
Dutch (nl)	15,806	2,895	5,195	13,344	2,616	3,941

#### Network configurations

We leverage the cased mBERT (https://github.com/google-research/bert/blob/master/multilingual.md) and XLM (https://dl.fbaipublicfiles.com/XLM/mlm_17_1280.pth) for the pretrained word representations, where the first pretrained language model includes 12 Transformer blocks, 768 hidden units, 12 self-attention heads, and the second one is with 16 Transformer blocks, 1028 hidden units, 16 self-attention heads. When adapters are applied, the dimensions of middle vectors are all set to 256. The language embedding size is set to 16. For the BiLSTM encoding part, the hidden size is set to 200.

#### Network training

We implement all our proposed models based on the huggingface Transformers (https://github.com/huggingface/transformers). We use online batch learning to optimize model parameters, where the batch size is set to 32. For the optimizer, we adopt AdamW with a learning rate of 5e-4 [25]. Furthermore, we use gradient normalization as well by a max norm of 5. We train each model for several iterations, and use the model with the best performance on the development set as the final model.

#### Offline learning of language embeddings

In particular, our PGN network requires offline language embeddings since there is no way to connect the target language embeddings with the source language embeddings in our unsupervised setting. As mentioned in the Model section before, we achieve the goal by a binary classification framework to judge whether two sentences of different languages are with the same meaning. We exploit the Europarl V7 (https://www.statmt.org/europarl/) to obtain the parallel corpora as the positive instances, and the negative examples are limited to the sentence set of these parallel corpora. We sample randomly by a ratio of 1:5 corresponding to positive v.s. negative. In addition, give the representations of a paired sentence, we exploit the absolute value function over their vector-subtraction for feature extraction, which is used for the next binary classification.

#### Evaluation

We use entity-level F1-score as the primary evaluation metric, following previous NER studies [5], and precision (P) and recall (R) are also reported. For each experiment, we conduct 5 runs and report the average F1-score.

### Main results

#### Single-source evaluations

Table 2 reports the main results of single-source cross-lingual NER based on different feature-extraction methods and pretrained language models. There are several interesting findings.

**Table 2:** Main results of single-source cross-lingual NER, where lavg indicates the averaged performance for each target language, and avg denotes the overall average F-scores of all source-target pairs.

Target	Source (mBERT)						Source (XLM)					
	en	de	es	nl	lavg	avg	en	de	es	nl	lavg	avg
Fine-Tuning												
en	-	72.96	73.62	**76.33**	74.30	72.61	-	73.32	72.98	**78.33**	74.88	73.27
de	70.16	-	66.79	**71.09**	69.35		68.52	-	66.07	**71.16**	68.58	
es	**75.42**	75.05	-	72.77	74.41		76.71	75.67	-	**78.45**	76.94	
nl	**77.45**	68.16	71.51	-	72.37		**79.08**	67.68	71.29	-	72.68	
Feature-based												
en	-	73.81	73.45	**77.25**	74.84	73.31	-	74.85	72.03	**78.34**	75.07	73.87
de	70.23	-	68.72	**71.12**	70.02		69.27	-	67.10	**71.85**	69.41	
es	**77.04**	74.27	-	72.93	74.75		**77.95**	76.86	-	77.93	77.58	
nl	**77.96**	70.86	72.13	-	73.65		**79.25**	68.08	72.92	-	73.42	
Feature-based (PGN)												
en	-	74.98	74.59	**77.59**	75.72	**73.99**	-	75.78	75.12	**78.94**	76.61	**74.96**
de	70.31	-	70.03	**71.26**	70.53		69.52	-	69.07	**72.03**	70.21	
es	**77.59**	74.46	-	73.28	75.11		78.01	76.93	-	**78.87**	77.94	
nl	**78.01**	72.15	73.62	-	74.59		**79.30**	72.09	73.87	-	75.09	

First, the English and Dutch as the sources languages can achieve better performances for cross-lingual transfer. The potential reason is complicated, which includes at least three aspects: the size of the training corpus, the size and distribution of the training entities, and the distances between the source and target languages. As shown, we can see that the training corpus size might be more critical for the transfer.

Second, the XLM as the pretrained word representations brings better performance than mBERT as a whole. Under the fine-tuning, feature-based as well as the feature-based (PGN) settings, the averaged improvements are 73.27 − 72.61 = 0.66, 73.87 − 73.31 = 0.56, 74.96 − 73.99 = 0.97, respectively.

Third, our feature-based method is better than the standard fine-tuning method. The observation indicates that the feature-based method is a better alternative in both parameter efficiency and performance. We will see that the feature-based method only consumes one-thirteenth of the parameters of the fine-tuning method.

Finally, the feature-based model with PGN can capture the language relationship effectively, resulting in significantly better performance in cross-lingual transfer as a whole. Notice that the exploration of PGN might be not as significant (i.e., the improvements larger than 0.5% can be regarded as significant by pairwise t-test below 10^−4^) in some of the language pair transfers. However, PGN is more desirable since it is more robust, which seldom gives degraded performances for all these language pairs. The effectiveness of PGN has been demonstrated in several other tasks, and our observation is consistent with theirs. Therefore, according to the results, we adopt the feature-based XLM model combined with PGN as the most preferred selection for the cross-lingual NER transfer.

#### Multi-source evaluations

Further, we perform multi-source cross-lingual transfer experiments. The leave-one-out manner is adopted to select source languages, i.e., all languages except the target one are regarded as source languages for multi-source transfer. Table 3 reports the results of different methods for multi-source cross-lingual NER. We investigate the multi-source transfer by the same strategies as the single-source transfer, and also, the best-reported results of the single-source transfer are used for comparisons.

**Table 3:** Main results of multi-source cross-lingual NER, where all other languages except the target language itself are exploited as the source languages.

Model	Target				Average
	en	de	es	nl	
mBERT					
Fine-Tuning	77.92	72.35	77.91	79.03	76.80
Feature-based	78.25	73.16	78.29	79.82	77.38
Feature-based (PGN)	**79.94**	**73.21**	**78.37**	**80.03**	**77.89**
Single-Source (Best)	77.59	71.26	77.59	78.01	74.11
XLM					
Fine-Tuning	79.89	71.12	79.79	77.93	77.43
Feature-based	79.97	**72.87**	80.35	79.76	78.49
Feature-based (PGN)	**80.67**	72.86	**80.95**	**80.88**	**79.09**
Single-Source (Best)	78.94	72.03	78.87	79.30	77.29

By examining the results of various multi-source cross-lingual transfer models, we can see that for each pretrained language model, feature-based adapter models outperform the standard fine-tuning methods. In addition, PGN can further advance the performance of multi-source cross-lingual transfer in most cases, and the overall averaged performance can be boosted by $\frac{0.61+0.6}{2}=0.61$ for mBERT and XLM. The observation is similar to that of single-source transfer. Although PGN is not able to give significantly better performance in all scenarios, it is a better choice since it can seldom degrade the transfer performance (i.e., only one exception by 72.86 v.s. 72.87). By comparing mBERT and XLM, we can see that XLM is better. Both observations are consistent with those of the single-source transfer, which further demonstrate the effectiveness of feature-based PGN strategy and the exploration of XLM.

Finally, we compare the results of multi-source cross-lingual transfer with single-source transfer. As shown, the multi-source transfer can give significantly better performance in all languages. The overall increments are 77.89 − 74.11 = 3.78 and 79.09 − 77.29 = 1.80 for mBERT and XLM, respectively.

#### Comparisons with previous work

Here we compare our proposed models with the previous state-of-the-art methods. For fair comparisons, we take English as the source language and German, Spanish, and Dutch as the target languages for the single-source model transfer. Table 4 reports the results of different methods for single-source and multi-source cross-lingual NER. We list the results of our three models based on XLM. It can be seen that our methods are comparable to the previous state-of-the-art methods. Remarkably, the feature-based model with PGN achieves highly-competitive performances, obtaining the best F1-values in the Spanish language. Moreover, compared with the best method (Wu et al., 2020) [29], which fully exploits both the unlabeled corpus of the target language and the labeled corpus of the source language, our feature-based models are only trained on source language and require much fewer model parameters and computational costs for both training and inference, meanwhile reaching comparable performances.

**Table 4:** Comparisons with previous studies.

Model	Target			Average
	de	es	nl	
Single-Source				
Xie et al. (2018) [26]	72.37	71.25	57.76	67.13
Moon et al. (2019) [27]	71.42	75.67	80.38	75.82
Wu and Dredze (2019) [28]	71.10	74.50	79.50	75.03
Wu et al. (2020) [29]	**73.16**	76.75	**80.44**	**76.78**
Fine-Tuning	68.52	76.71	79.08	74.77
Feature-based	69.27	77.95	79.25	75.49
Feature-based (PGN)	69.52	**78.01**	79.30	75.61
Multi-Source				
Täckström (2012) [30]	36.40	61.90	59.90	52.73
Rahimi et al. (2019) [31]	59.10	71.80	67.60	66.17
Chen et al. (2019) [32]	56.00	73.50	72.40	67.30
Moon et al. (2019) [27]	72.44	76.53	**83.35**	77.44
Wu et al. (2020b)* [33]	**81.33**	75.33	78.00	**78.22**
Fine-Tuning	71.12	79.79	77.93	76.28
Feature-based	72.87	80.35	79.76	77.66
Feature-based (PGN)	72.86	**80.95**	80.58	78.13

### Analysis

#### mBERT v.s. XLM

Our main experiments show that XLM can lead to better performance than mBERT in most cases. Considering that mBERT is pretrained simply by concatenating all corpora of different languages directly, while XLM leverages additional parallel corpora of different language pairs as well, the advantage of XLM over mBERT is reasonable, which also indicates the great value of these parallel corpora.

**Fig 4:**
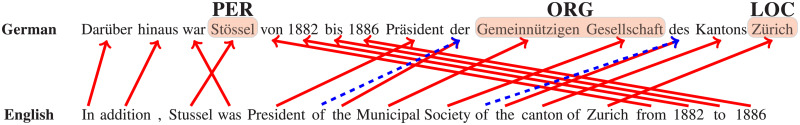
An example of word alignment visualization between a German sentence and its English translation, where the solid arrows are gold-standard being all correctly predicted by XLM, and the dashed arrows are incorrectly aligned by mBERT, and the others are the same for the two types of word representations.

In order to understand the advantage of XML clearly, we offer one example to compare the word-level alignments between XLM and mBERT by a parallel sentence with named entities inside. Intuitively, word alignments can be a good visualizing indicator to demonstrate the transferability of the multilingual word representations. The calculation of word alignments is performed straightforwardly by using the cosine scores between the vectorial representations of pairwise sentential words, where the one with the highest cosine score is chosen as the alignment. Here we only perform one-side alignment, which is shown in Fig 4. As expected, XLM can give an overall better alignment quality, which could guide NER implicitly. The success of XLM indicates that more sophisticated multilingual word representations with certain supervision can bring more gains for cross-lingual NER transfer.

#### The advantage of adapter

Our feature-based models exploit adapters to extract features from the pretrained transformer-based language model, where the extracted features are used as the basic word representations. As claimed, the method is much more parameter than the widely-adopted fine-tuning architecture for the BERT and XLM language models. Here we analyze the two strategies in detail. The results are shown in Table 5, where both the single-source and multi-source transfers are reported, and XLM is exploited as the input backbone for the discussion. We consider a gradual way to insert the adapter modules from the top transfer layers incrementally to the bottom transformer layers, i.e., the covered transformer layers by adapter from zero to all 16 layers.

**Table 5:** The comparisons between the fine-tuning and feature-based adapter exploration methods, where XLM is used as the input language model.

Model	Single-Source	Multi-Source	Trainable Params Size
Fine-Tuning	73.27	77.43	**230M**
The feature-based method with adapter inside			
0 layers	71.83	75.81	0.6M
2 layers	72.68	77.00	2.7M
4 layers	73.16	77.45	4.8M
6 layers	73.44	77.81	6.9M
8 layers	73.66	78.31	9.0M
10 layers	73.68	78.44	11.1M
12 layers	73.82	78.33	13.2M
14 layers	**74.05**	78.40	15.3M
16 layers (all)	73.87	**78.49**	17.4M

As shown, it is apparent that XLM with Adapter is much more parameter efficient, and even when all layers are exploited, the model consumes below one-thirteenth parameter numbers compared with BERT fine-tuning. The straightforward feature-based method without using adapter performs worse than XLM fine-tuning, which indicates the importance of adapter. As the number of covered transformers increases, the performance is gradually boosted, and after 4 layers, the performance is comparable with XLM fine-tuning. Finally, we select the model by applying full 16 layers with adapters since it can achieve the best overall performance.

#### The effect of source-target languages

**Fig 5:**
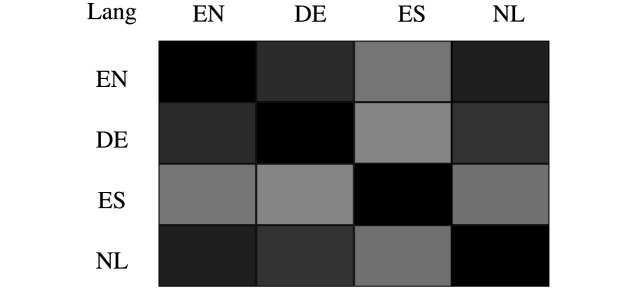
The similarity heatmap of language embeddings for different language pairs, where deeper color indicates higher similarity.

Further, we examine the effect of model transfer with respect to different source-target language pairs. Fig 5 shows the heatmap matrix for different language pairs, which are computed according to the pretrained language embeddings. As shown, we can find that the English, German and Dutch languages are highly similar, while the Spanish language is slightly away from the three languages. The observation is reasonable since the English, German and Dutch languages all belong to the Germanic branch of the Indo-European language family, and the Spanish language is from the Italic branch.

Further, we investigate how source-target languages influence the *PGN* model. Intuitively, language pairs with larger differences can benefit more from the PGN module. As shown in Table 2, we find that indeed es-other and other-es can obtain more improvements by PGN, which is consistent with our intuition.

#### Case study

Finally, we offer a case study to compare the cross-lingual model transfer methods. We focus on the comparisons between the single-source transfer without PGN and with PGN, as well as the multi-source transfer without PGN and with PGN. All models exploit XLM as the backbone, and the feature-based method is selected used. As shown in Table 6, we can see that PGN can help to obtain more accurate entity boundaries, while multi-source transfer can recall more named entities.

**Table 6:** A case study, where the text with underlines indicates errors.

Model	Text and Entities
**Single-Source (Dutch-English)**	
Feature-Based	Australian parliamentarian [**John Langmore**]_*PER*_ has formally resigned from his lower house seat, [**the office of House of Representatives speaker**]_*ORG*_ [**Bob Halverson**]_*PER*_ said on Friday.
Feature-Based (PGN)	Australian parliamentarian [**John Langmore**]_*PER*_ has formally resigned from his lower house seat, the office of [**House of Representatives**]_*ORG*_ speaker [**Bob Halverson**]_*PER*_ said on Friday.
**Multi-Source (Others-English)**	
Feature-Based	[**Australian parliamentarian**]_*LOC*_ [**John Langmore**]_*PER*_ has formally resigned from his lower house seat, [**the office of House of Representatives**]_*ORG*_ speaker [**Bob Halverson**]_*PER*_ said on Friday.
Feature-Based (PGN)	[**Australian**]_*MISC*_ parliamentarian [**John Langmore**]_*PER*_ has formally resigned from his lower house seat, the office of [**House of Representatives**]_*ORG*_ speaker [**Bob Halverson**]_*PER*_ said on Friday.
Ground-truth	[**Australian**]_*MISC*_ parliamentarian [**John Langmore**]_*PER*_ has formally resigned from his lower house seat, the office of [**House of Representatives**]_*ORG*_ speaker [**Bob Halverson**]_*PER*_ said on Friday.

## Related work

Here we introduce the related work by six aspects, including NER, cross-lingual NER, model transfer, multi-source cross-lingual transfer, adapter and PGN.

### NER

Early NER systems are based on handcrafted rules, lexicons, semantic and syntactic features. These systems are followed by statical machine learning models with careful feature-engineering of humans [1]. Recently, neural network models have become the dominant methods for NER due to their high performance [6, 34–36]. Especially, the pretrained contextualized word representations such as ELMO and BERT have advanced the NER performance greatly [37–40]. The NER system based on BERT together with CRF decoding can achieve state-of-the-art performance [6, 7]. Our basic model is built according to the system, and our work focuses on the unsupervised cross-lingual setting, studying different exploration methods for the BERT-alike word representations.

### Cross-lingual NER

Cross-lingual NER has been a hot topic in the NLP community [13, 41]. There are two mainstream categories for cross-lingual NER. The translation-based category aims to build pseudo-labeled data for a target language, and then the data is used to train a target NER model [26, 41–43]. The methods of this category always require a mount of parallel corpora (or a translation lexicon). In this work, we also exploit parallel corpora to pretrain language embeddings, while our method is highly different from theirs.

### Model transfer

Our work follows another line of cross-lingual NER, namely model transfer [13], which is quite simple and straightforward. The category has been studied intensively before for tasks such as parsing [11, 12]. Based on language-independent features such as cross-lingual word clusters [44], word embeddings [14] and gazetteers [45], a model trained on the source language can be directly applied to the target language. Here we exploit multilingual contextualized word representations [15, 16] and present several substantial improvements for the model transfer. This is the first work to study the unsupervised model transfer for NER comprehensively.

### Multi-source cross-lingual transfer

McDonald et al. (2011) [11] present the method of multi-source transfer for unsupervised cross-lingual adaption. They simply concatenate all labeled datasets of different source languages, which are used to train a unified model being able to handle the target language. Moon et al. (2019) [27] exploit the idea to cross-lingual NER. The two studies are both extended model transfer essentially, and treat all source languages equally. Chen et al. (2019) [32] leverage adversarial neural networks to weigh the importance of different source languages better, considering that different languages may contribute differently to a target language. In this work, we present a different method to model the different source languages.

### Adapter

The adapter has been originally investigated in the computer vision community, aiming to adapt a model for multiple domains [46]. Recently, the adapter modules have been applied to NLP for quickly adapting a pretrained transformer model to new domains and tasks without fine-tuning the transformer model [19, 20, 47], thus it is much parameter-efficient. Here we use the adapter modules to enhance the basic model for a parameter-efficient NER model, and it can be combined with PGN seamlessly for cross-lingual transfer.

### PGN

PGN is essentially used to disclose the relationship between tasks, domains and languages by embedding vectors, where the similarities and differences between different tasks, domains and languages can be both captured. Initially, Platanios et al. (2018) [17] learn language vectors and them to generate parameters for multilingual machine translation. Jia et al. (2019) [48] apply the idea firstly for multi-domain adaptation, using NER as the case. Fei et al. (2020) [49] adopt PGN to cross-lingual semantic role transfer. Ust un et al. (2020) [50] present the work most similarly to our work, which combines PGN and adapter for universal dependency parsing. However, they are not aimed for an unsupervised cross-lingual transfer, and they can learn language embeddings directly through the training data. Our work adopts a similar word representation method, but exploits a distant supervision method for language embedding learning.

## Conclusion and future work

In this work, we investigated the unsupervised cross-lingual adaption for NER based on the model transfer framework. We focused on the NER models based on contextualized word representations since they can benefit NER much and lead to a state-of-the-art system. We chosen two types of multilingual contextualized word representation, mBERT and XLM, respectively, comparing their performances by different exploration methods. In addition, we extended single-source model transfer to multi-source transfer as well, as the latter can bring better performance and meanwhile are more suitable for the real setting. We proposed a novel model with sophisticated neural networks to exploit multilingual word representations. Concretely, we applied the adapter mechanism to enhance the feature-based exploration method of the pretrained transformer language models, and further adopted PGN to better encode the relationship between different languages including the source and target languages. In order to learn effective language embeddings for PGN, we suggested a novel pretraining strategy by using parallel corpora of mixed language pairs.

Finally, we conducted experiments to verify the performance of various models. We selected a benchmark dataset from ConLL evaluation tasks for simulation evaluations, which includes four languages, including English, Spanish, Germany and Dutch. The results show that XLM can achieve better model transfer performance for different languages with various settings, due to its pretraining with cross-lingual supervision, which hints that multilingual word representations with rich supervised pretraining might be more prospective for the cross-lingual NER. Although fine-tuning can achieve impressive results, the adapter-enhanced feature-based models can be more prospective. In particular, the feature-based adapter model with PGN can greatly boost the final performance for unsupervised cross-lingual transfer.

The model transfer can still have great potentials for unsupervised cross-lingual transfer. For example, the method can be integrated with translation-based methods, where the language embeddings of PGN might be more effectively learned. In addition, there could exist more types of powerful multilingual word representations in the future, which can be exploited as inputs as well. These attempts can be served as future studies.

## References

[1] Nadeau D, Sekine S. A survey of named entity recognition and classification. Lingvisticae Investigationes. 2007;30(1):3–26. doi: 10.1075/li.30.1.03nad

[2] Lin Y, Shen S, Liu Z, Luan H, Sun M. Neural relation extraction with selective attention over instances. In: Proceedings of ACL; 2016. p. 2124–2133.

[3] Lee K, He L, Lewis M, Zettlemoyer L. End-to-end neural coreference resolution. In: Proceedings of EMNLP; 2017.

[4] Lukovnikov D, Fischer A, Lehmann J, Auer S. Neural network-based question answering over knowledge graphs on word and character level. In: Proceedings of WWW; 2017. p. 1211–1220.

[5] McCallum A, Li W. Early results for named entity recognition with conditional random fields, feature induction and web-enhanced lexicons. In: Proceedings of NAACL-HLT; 2003. p. 188–191.

[6] Luo Y, Xiao F, Zhao H. Hierarchical Contextualized Representation for Named Entity Recognition. In: Proceedings of AAAI; 2020.

[7] Cui L, Zhang Y. Hierarchically-Refined Label Attention Network for Sequence Labeling. In: Proceedings of EMNLP-IJCNLP; 2019.

[8] Devlin J, Chang MW, Lee K, Toutanova K. BERT: Pre-training of Deep Bidirectional Transformers for Language Understanding. In: NAACL-HLT (1); 2019.

[9] Mayhew S, Nitish G, Roth D. Robust named entity recognition with truecasing pretraining. In: Proceedings of the AAAI Conference on Artificial Intelligence. vol. 34; 2020. p. 8480–8487.

[10] Mengge X, Yu B, Zhang Z, Liu T, Zhang Y, Wang B. Coarse-to-Fine Pre-training for Named Entity Recognition. In: Proceedings of the EMNLP; 2020. p. 6345–6354.

[11] McDonald R, Petrov S, Hall K. Multi-Source Transfer of Delexicalized Dependency Parsers. In: Proceedings of the EMNLP; 2011. p. 62–72.

[12] Guo J, Che W, Yarowsky D, Wang H, Liu T. Cross-lingual dependency parsing based on distributed representations. In: Proceedings of the ACL; 2015. p. 1234–1244.

[13] Zirikly A, Hagiwara M. Cross-lingual transfer of named entity recognizers without parallel corpora. In: Proceedings of the 53rd ACL; 2015. p. 390–396.

[14] Huang L, Ji H, May J. Cross-lingual multi-level adversarial transfer to enhance low-resource name tagging. In: Proceedings of the NAACL; 2019. p. 3823–3833.

[15] Pires T, Schlinger E, Garrette D. How Multilingual is Multilingual BERT? In: Proceedings of the 57th ACL; 2019. p. 4996–5001.

[16] Conneau A, Khandelwal K, Goyal N, Chaudhary V, Wenzek G, Guzmán F, et al. Unsupervised Cross-lingual Representation Learning at Scale. In: Proceedings of the 58th ACL; 2020. p. 8440–8451.

[17] Platanios EA, Sachan M, Neubig G, Mitchell T. Contextual Parameter Generation for Universal Neural Machine Translation. In: Proceedings of the EMNLP; 2018. p. 425–435.

[18] Vaswani A, Shazeer N, Parmar N, Uszkoreit J, Jones L, Gomez AN, et al. Attention is all you need. In: Advances in neural information processing systems; 2017. p. 5998–6008.

[19] Stickland AC, Murray I. BERT and PALs: Projected attention layers for efficient adaptation in multi-task learning. In: International Conference on Machine Learning. PMLR; 2019. p. 5986–5995.

[20] Houlsby N, Giurgiu A, Jastrzebski S, Morrone B, De Laroussilhe Q, Gesmundo A, et al. Parameter-efficient transfer learning for NLP. In: International Conference on Machine Learning. PMLR; 2019. p. 2790–2799.

[21] Hochreiter S, Schmidhuber J. Long short-term memory. Neural computation. 1997;9(8):1735–1780. doi: 10.1162/neco.1997.9.8.1735 9377276

[22] Lafferty JD, McCallum A, Pereira FCN. Conditional Random Fields: Probabilistic Models for Segmenting and Labeling Sequence Data. In: Proceedings of the ICML; 2001. p. 282–289.

[23] Tjong Kim Sang EF. Introduction to the CoNLL-2002 shared task: language-independent named entity recognition. In: Proceedings of the CoNLL; 2002. p. 1–4.

[24] Sang ETK, De Meulder F. Introduction to the CoNLL-2003 Shared Task: Language-Independent Named Entity Recognition. In: Proceedings of CoNLL-2003; 2003. p. 142–145.

[25] Loshchilov I, Hutter F. Decoupled Weight Decay Regularization. In: International Conference on Learning Representations; 2018.

[26] Xie J, Yang Z, Neubig G, Smith NA, Carbonell JG. Neural Cross-Lingual Named Entity Recognition with Minimal Resources. In: Proceedings of the EMNLP; 2018. p. 369–379.

[27] Moon T, Awasthy P, Ni J, Florian R. Towards lingua franca named entity recognition with bert. arXiv preprint arXiv:191201389. 2019;.

[28] Wu S, Dredze M. Beto, bentz, becas: The surprising cross-lingual effectiveness of bert. In: Proceedings of EMNLP-IJCNLP; 2019. p. 833–844.

[29] Wu Q, Lin Z, Wang G, Chen H, Karlsson BF, Huang B, et al. Enhanced meta-learning for cross-lingual named entity recognition with minimal resources. In: Proceedings of the AAAI Conference on Artificial Intelligence. vol. 34; 2020. p. 9274–9281.

[30] Täckström O. Nudging the envelope of direct transfer methods for multilingual named entity recognition. In: NAACL-HLT 2012 Workshop on Inducing Linguistic Structure; 2012.

[31] Rahimi A, Li Y, Cohn T. Massively Multilingual Transfer for NER. In: Proceedings of the 57th Annual Meeting of the Association for Computational Linguistics; 2019. p. 151–164.

[32] Chen X, Hassan A, Hassan H, Wang W, Cardie C. Multi-Source Cross-Lingual Model Transfer: Learning What to Share. In: Proceedings of the 57th ACL; 2019. p. 3098–3112.

[33] Wu Q, Lin Z, Karlsson B, Jian-Guang L, Huang B. Single-/Multi-Source Cross-Lingual NER via Teacher-Student Learning on Unlabeled Data in Target Language. In: Proceedings of the 58th Annual Meeting of the Association for Computational Linguistics; 2020. p. 6505–6514.

[34] Huang Z, Xu W, Yu K. Bidirectional LSTM-CRF models for sequence tagging. arXiv preprint arXiv:150801991. 2015;.

[35] Lample G, Ballesteros M, Subramanian S, Kawakami K, Dyer C. Neural Architectures for Named Entity Recognition. In: Proceedings of NAACL-HLT; 2016. p. 260–270.

[36] Luo Y, Zhao H, Zhang Z, Tang B. Open Named Entity Modeling from Embedding Distribution. IEEE Transactions on Knowledge and Data Engineering. 2021;.

[37] Akbik A, Blythe D, Vollgraf R. Contextual string embeddings for sequence labeling. In: Proceedings of COLING; 2018. p. 1638–1649.

[38] Devlin J, Chang MW, Lee K, Toutanova K. Bert: Pre-training of deep bidirectional transformers for language understanding. In: Proceedings of NAACL-HLT; 2018.

[39] Li X, Feng J, Meng Y, Han Q, Wu F, Li J. A Unified MRC Framework for Named Entity Recognition. In: Proceedings of ACL; 2020. p. 5849–5859.

[40] Straková J, Straka M, Hajič J. Neural architectures for nested NER through linearization. 2019; p. 5326–5331.

[41] Wang M, Manning CD. Cross-lingual projected expectation regularization for weakly supervised learning. Transactions of the Association for Computational Linguistics. 2014;2:55–66.

[42] Ni J, Dinu G, Florian R. Weakly Supervised Cross-Lingual Named Entity Recognition via Effective Annotation and Representation Projection. In: Proceedings of the 55th ACL; 2017. p. 1470–1480.

[43] Mayhew S, Tsai CT, Roth D. Cheap translation for cross-lingual named entity recognition. In: Proceedings of the 2017 EMNLP; 2017. p. 2536–2545.

[44] Täckström O, McDonald R, Uszkoreit J. Cross-lingual word clusters for direct transfer of linguistic structure. In: Proceedings of NAACL; 2012.

[45] Tsai CT, Mayhew S, Roth D. Cross-lingual named entity recognition via wikification. In: Proceedings of The 20th SIGNLL Conference on Computational Natural Language Learning; 2016. p. 219–228.

[46] Rebuffi SA, Bilen H, Vedaldi A. Efficient parametrization of multi-domain deep neural networks. In: Proceedings of the IEEE Conference on CVPR; 2018. p. 8119–8127.

[47] Pfeiffer J, Vulić I, Gurevych I, Ruder S. MAD-X: An Adapter-based Framework for Multi-task Cross-lingual Transfer. In: Proceedings of the EMNLP; 2020. p. 7654–7673.

[48] Jia C, Liang X, Zhang Y. Cross-domain NER using cross-domain language modeling. In: Proceedings of the 57th ACL; 2019. p. 2464–2474.

[49] Fei H, Zhang M, Ji D. Cross-Lingual Semantic Role Labeling with High-Quality Translated Training Corpus. In: Proceedings of the 58th ACL; 2020. p. 7014–7026.

[50] Üstün A, Bisazza A, Bouma G, van Noord G. UDapter: Language Adaptation for Truly Universal Dependency Parsing. In: Proceedings of the EMNLP; 2020. p. 2302–2315.

